# Intracameral Sustained-Release Bimatoprost Implant Delivers Bimatoprost to Target Tissues with Reduced Drug Exposure to Off-Target Tissues

**DOI:** 10.1089/jop.2018.0067

**Published:** 2019-01-29

**Authors:** Jennifer R. Seal, Michael R. Robinson, James Burke, Marina Bejanian, Michael Coote, Mayssa Attar

**Affiliations:** ^1^ Allergan plc, Irvine, California.; ^2^ Center for Eye Research Australia, Royal Victorian Eye and Ear Hospital, Melbourne, Australia.

**Keywords:** bimatoprost, eye drops, implant, ocular drug distribution, pharmacokinetics, preclinical

## Abstract

***Purpose:*** To explore the ocular distribution of bimatoprost after intracameral administration of a biodegradable sustained-release bimatoprost implant (Bimatoprost SR) versus repeated topical administration of bimatoprost 0.03% ophthalmic solution in dogs. Bimatoprost SR and topical bimatoprost 0.03% previously were shown to have similar intraocular pressure-lowering effects in humans in a phase 1/2 clinical trial.

***Methods:*** Twenty-four beagle dogs received either once-daily topical bimatoprost 0.03% for 7 days or a bilateral intracameral administration of Bimatoprost SR (15 μg). At predetermined time points, ocular tissues were collected and concentrations of bimatoprost and bimatoprost acid were quantified using liquid chromatography–tandem mass spectrometry.

***Results:*** Bimatoprost SR administration enhanced delivery of study drug to a site of action [iris–ciliary body (ICB)] compared with topical bimatoprost (*C*_max_ [bimatoprost+bimatoprost acid] = 18,200 and 4.13 ng/g, respectively). However, distribution of drug to tissues associated with prostaglandin analog (PGA)-related side effects (i.e., bulbar conjunctiva, eyelid margins, and periorbital fat) was limited following Bimatoprost SR administration (*C*_max_ [bimatoprost+bimatoprost acid] = BLQ [beneath the limit of quantitation] to 0.354 ng/g) compared with topical dosing (*C*_max_ [bimatoprost+bimatoprost acid] = 36.6–2,110 ng/g).

***Conclusions:*** Bimatoprost SR administration in dogs selectively delivered drug to the ICB with low or undetectable drug levels in ocular surface and extraocular tissues. Use of Bimatoprost SR for glaucoma treatment may reduce the incidence of adverse events typically associated with topical PGAs by targeting bimatoprost delivery to the key site of action of the PGA class and reducing exposure to off-target tissues.

## Introduction

Glaucoma, a group of ocular disorders with a multifactorial etiology, is characterized by the presence of intraocular pressure (IOP)-associated optic neuropathy.^1^ Glaucoma affects an estimated 60.5 million people worldwide and is the second leading cause of blindness (after cataract).^2,3^ The most common type of glaucoma, open-angle glaucoma, is a chronic, progressive disease resulting in loss of retinal ganglion cells, optic nerve atrophy, and irreversible vision loss.^4^

The principal goal of glaucoma treatment is to reduce IOP and preserve visual function while maintaining patient quality of life.^5^ IOP can be lowered with pharmacological treatments, laser treatments, and various minimally invasive or incisional surgical procedures. Pharmacological therapies are currently the most common form of initial intervention. Topical prostaglandin analog (PGA) medications (such as latanoprost, tafluprost, travoprost, and the prostamide bimatoprost) are widely used as first-line therapies due to their proven efficacy, favorable safety profile, and convenient once-daily dosing schedule.^5–9^

Despite the widespread use of topical eye drops for IOP lowering, patient compliance with the prescribed administration regimen, especially when multiple drops are needed daily, is a critical issue.^10^ Given the fact that the majority of glaucoma cases are asymptomatic until the late stages of the disease,^5^ patients may be less inclined to administer their medication on a regular or continued basis.^11^ Reasons for nonadherence also include forgetfulness, difficulty in instilling eye drops, dosing frequency, medication cost, and side effects.^12–14^ Conjunctival hyperemia, eyelash growth, iris and periocular skin pigmentation, and rarely, cystoid macula edema and periorbitopathy are among the side effects that have been noted following PGA use.^15–18^

Sustained-release formulations are currently being developed to circumvent some of the limitations associated with topical medications. These formulations are intended to provide prolonged drug exposure to target tissues without the need for daily administration, with the expectation of improving adherence to therapy.^19,20^ In addition, use of intraocular sustained-release formulations has the potential to reduce the occurrence of ocular surface and periocular adverse events (AEs) associated with topical administration.^20^

**FIG. 1.**
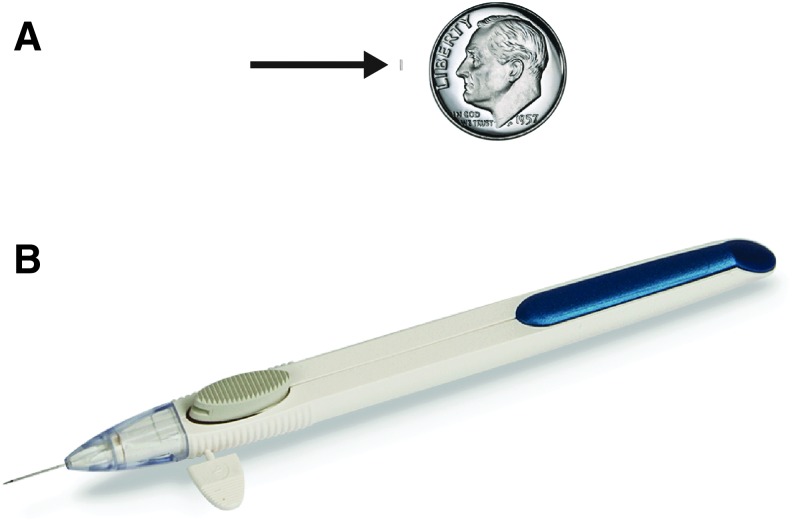
Photographs of the sustained-release bimatoprost implant (Bimatoprost SR, *arrow*) next to a dime for size comparison **(A)** and the ready-to-use applicator **(B)**.

Bimatoprost sustained-release implant (Bimatoprost SR, Fig. 1A) is a biodegradable implant that is currently in clinical development. The solid, rod-shaped implant consists of bimatoprost within the biodegradable NOVADUR platform for drug delivery^19^ (Allergan plc, Dublin, Ireland). For Bimatoprost SR, the NOVADUR platform was modified to release bimatoprost with nonpulsatile, steady-state drug release (i.e., zero-order kinetics).^20^ The intracamerally placed implant was designed to provide slow release of bimatoprost to lower IOP in patients with glaucoma for 4–6 months. As the implant is placed in the intracameral space, it is anticipated to target drug delivery directly to the key site of action of the PGA class, the iris–ciliary body (ICB), and may reduce the AEs associated with topical application of PGAs by limiting drug distribution to the conjunctiva and periocular tissues. This article describes the distribution of bimatoprost in ocular tissues of beagle dogs following bilateral administration of either a topical bimatoprost 0.03% ophthalmic solution or Bimatoprost SR containing 15 μg of bimatoprost (Bimatoprost SR 15 μg). In humans, daily treatment with topical bimatoprost 0.03% and a single administration of Bimatoprost SR 15 μg have been shown to produce comparable overall reductions in IOP through week 16.^20^

## Methods

### Animals

Twenty-four female beagle dogs (Marshall BioResources, New York, NY and Covance, VA), ages 18 months to 3 years, weighing 7–12 kg, were housed under controlled conditions. Animals received a certified canine diet once daily and drinking water was provided *ad libitum*. All procedures were performed in compliance with the United States Department of Agriculture (USDA), the U.S. Animal Welfare Act, and Allergan's Animal Care and Use Committee (AACUC) guidelines and were consistent with the Association for Research in Vision and Ophthalmology (ARVO) Statement for the Use of Animals in Ophthalmic and Vision Research.

### Animal screening and assignment

Dogs were assigned to 2 treatment groups based on iridocorneal angle data. Anterior segment optical coherence tomography (AS-OCT; Visante™ OCT, St. Paul, MN) was performed on both eyes of each animal to determine the iridocorneal angle size. It has been previously reported that latanoprost can cause miosis and narrowing of the iridocorneal angle in dogs.^21^ Topical bimatoprost 0.03% was administered, and after ∼30 min, animals were anesthetized with an intravenous (IV) cocktail of ketamine (5–8 mg/kg), xylazine (0.5–0.8 mg/kg), and acepromazine (0.1–0.175 mg/kg). Images were taken from the temporal quadrant using regular or enhanced anterior segment single eye scans. When it was not possible to image the temporal area, an image of the inferotemporal or superior quadrant was used. All images bisected the pupil. The iridocorneal angle size was determined from AS-OCT images by measuring the largest phantom circle that fit within the iridocorneal angle with a point of contact just behind Schwalbe's line. Animals were grouped according to their iridocorneal angle size; animals with larger iridocorneal angles (≥344 μm) were included in the Bimatoprost SR 15 μg treatment group to minimize the possibility of implant contact with the corneal endothelium. Animals with angles <344 μm were included in the topical bimatoprost 0.03% treatment group. A 2-week washout period followed the imaging procedure to ensure that the animals were no longer exposed to topical bimatoprost.

### Bimatoprost administration

Topical bimatoprost 0.03% ophthalmic solution (Lumigan^®^; Allergan plc, Dublin, Ireland) was supplied in ready-to-use dropper bottles and Bimatoprost SR 15 μg was supplied in a single-use, ready-to-use applicator (Fig. 1B) by the Pharmaceutical Science Operation Department at Allergan plc (Dublin, Ireland). Both products were stored at room temperature before dosing.

**FIG. 2.**
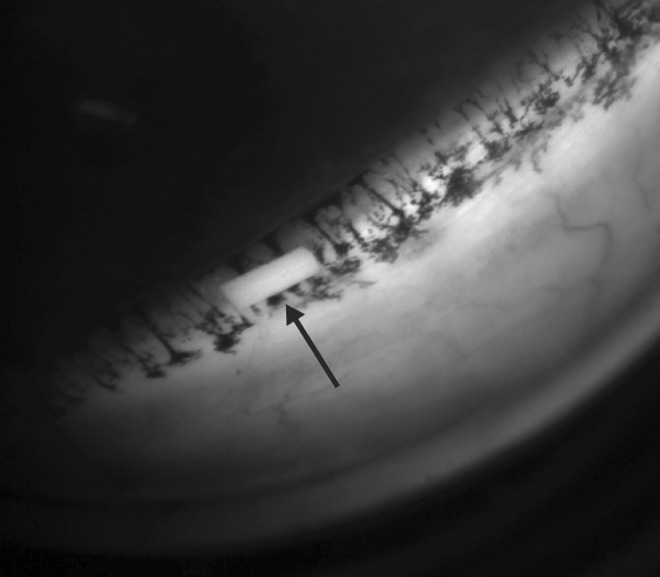
Gonioscopic image of bimatoprost sustained-release implant (Bimatoprost SR, *arrow*) nestled in the inferior angle of a beagle dog eye 1 week after dosing.

Animals with a normal ophthalmic examination were used. Ten animals received 1 drop (∼35 μL) of topical bimatoprost 0.03% ophthalmic solution once daily in each eye for 7 days (approximately the same time each day). Following eye drop administration, eyes were gently held closed for ∼5 s to ensure uniform dose distribution around the eye. Fourteen animals with no visible signs of eye abnormalities were prepared for intracameral administration by applying 1 drop of gatifloxacin (Zymar^®^; Allergan plc) twice to each eye, with ∼3 h between doses. After fasting overnight, animals were anesthetized as described above. Following instillation of 1–2 drops of ophthalmic anesthetic (proparacaine 0.5% or lidocaine 2%) to each eye, a 5% povidone–iodine ophthalmic solution (Betadine^®^; Alcon) was used to clean the periorbital area, including the eyelids, and bathe the conjunctival fornices and injection site. The solution remained on the eyes for 2–3 min; subsequently, each eye was irrigated with a sterile saline solution until the povidone–iodine solution was completely washed off. If required, 1–2 additional drops of ophthalmic anesthetic were applied to each eye before implantation of Bimatoprost SR 15 μg. The implant was injected into the anterior chamber using the sterile, preloaded applicator. After the procedure, 2 drops of gatifloxacin were administered to both eyes. Following injection, the implant generally resided in the inferior angle of the dog eye (Fig. 2).

### Sample collection

For the topical bimatoprost 0.03% group, animals were sacrificed on day 7 at 0.5, 1, 2, 4, and 9 h postdose (2 animals per time point). For the Bimatoprost SR 15 μg group, animals were sacrificed 1, 2, 3, 4.5, and 6 months after implant administration (2 animals per time point). Following euthanasia by IV pentobarbital 120 mg/kg (Euthasol^®^; Virbac USA, Fort Worth, TX) as per the AACUC protocol, eyes were enucleated and the aqueous humor, eyelid margins (upper and lower collected separately), periorbital fat, and bulbar conjunctiva were collected and weighed. The eye was subsequently flash frozen using liquid nitrogen and stored at −20°C. The ICB, retina (area centralis region, corresponding to the macular region in human eyes), and cornea, as well as any residual implant (if applicable) were collected and weighed within 2 days of freezing. All samples were stored at or below −70°C until analysis.

### Bioanalysis

Tissue extraction was achieved by overnight soaking in 75:25 methanol:water, with the exception of aqueous humor, where liquid–liquid extraction with methyl tert-butyl ether was employed. Sample analysis was conducted using a liquid chromatography–tandem mass spectrometer (API 5500 Q-trap; Sciex, Framingham, MA), an Acquity UPLC BEH C18 column (2.1 × 50 mm, 1.7 μm; Waters, Milford, MA), and gradient elution with mobile phases (A) ammonium bicarbonate (1 mM, pH 8) and (B) acetonitrile:methanol (1:1 vol/vol). The precursor–product ion pairs used for quantitation in multiple reaction monitoring mode (positive mode for bimatoprost, negative mode for bimatoprost acid) were: m/z 416 → 362 (bimatoprost), m/z 420 → 366 (bimatoprost-d4), m/z 387 → 192 (bimatoprost acid), and m/z 391 → 197 (bimatoprost acid-d4). The lower limit of quantitation (LLOQ) for bimatoprost and bimatoprost acid was ≤0.1 ng/mL for all ocular tissues. Implants were analyzed using high-performance liquid chromatography with an LLOQ for bimatoprost of 0.1 μg/mL.

### Pharmacokinetic and statistical analysis

A noncompartmental model was constructed using Phoenix WinNonlin 6.3 software (Certara, Princeton, NJ). Using the ocular tissues, the following pharmacokinetic parameters were calculated for bimatoprost and bimatoprost acid after each dose: maximal observed concentration (*C*_max_), time corresponding to maximal observed concentration (*T*_max_), and the area under the concentration–time curve from time zero to the last measurable time point (AUC_0-tlast_) calculated by the linear trapezoidal rule.

## Results

### Release of bimatoprost from implant in dog eye

Analysis of the Bimatoprost SR 15 μg remnants found that 80.5% of the bimatoprost load had been released by day 51. At the same time point, ocular tissue concentrations were generally at their maximum. By day 80, 99.8% of the drug load had been released, and there was a significant decline in ocular tissue concentrations.

### Ocular distribution of bimatoprost

**FIG. 3.**
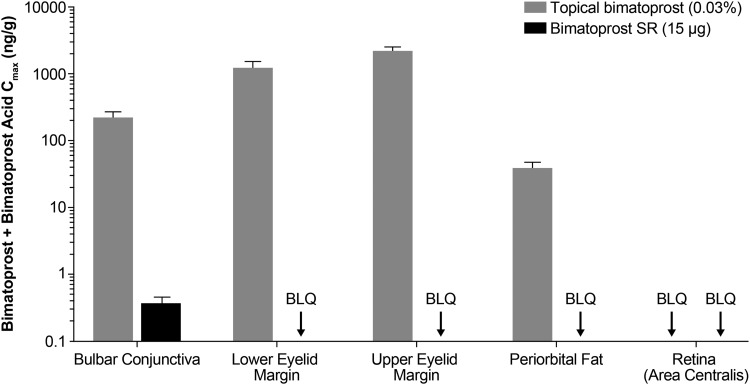
Mean bimatoprost plus bimatoprost acid *C*_max_ in the ocular tissues associated with PGA-related AEs following administration of either topical bimatoprost 0.03% once daily for 7 days or Bimatoprost SR 15 μg to beagle dogs. *n* = 2 animals/4 eyes per time point. AE, adverse event; Bimatoprost SR, bimatoprost sustained-release implant; BLQ, below the limit of quantitation; *C*_max_, maximal observed concentration; PGA, prostaglandin analog.

**FIG. 4.**
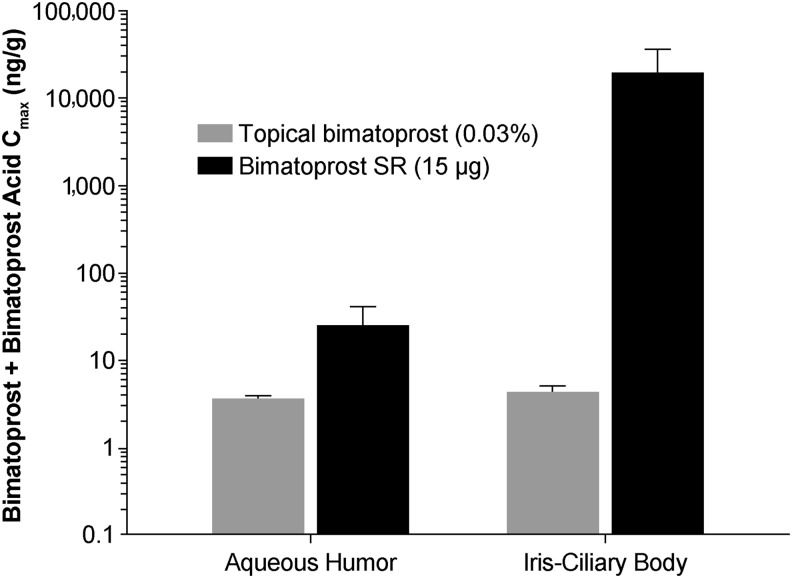
Mean bimatoprost plus bimatoprost acid *C*_max_ in the ocular tissues associated with efficacy following administration of either topical bimatoprost 0.03% once daily for 7 days or Bimatoprost SR 15 μg to beagle dogs. *n* = 2 animals/4 eyes per time point. Bimatoprost SR, bimatoprost sustained-release implant; *C*_max_, maximal observed concentration.

Following 7 days of once-daily administration of topical bimatoprost 0.03%, bimatoprost and bimatoprost acid had distributed to all tissues examined, except the area centralis of the retina (Table 1). The rank order of bimatoprost exposure was as follows: upper eyelid margin > lower eyelid margin > bulbar conjunctiva > periorbital fat > cornea > ICB > aqueous humor. The sum of bimatoprost and bimatoprost acid exposure in a purported site of pharmacologic action, the ICB, was 10- to 320-fold lower than in the ocular surface tissues (cornea, bulbar conjunctiva, and eyelid margins) (Figs. 3 and 4). Bimatoprost acid concentrations were higher than bimatoprost concentrations in the cornea, bulbar conjunctiva, aqueous humor, and ICB, reflecting a high rate of metabolism in these tissues. In contrast, very little metabolic conversion of bimatoprost to bimatoprost acid was observed in the eyelid margins or periorbital fat.

**Table 1.** Pharmacokinetic Parameters of (A) Bimatoprost and (B) Bimatoprost Acid in Ocular Tissues Following the Administration of Topical Bimatoprost 0.03% Once Daily for 7 Days to Beagle Dogs

*Ocular tissue*	C*_max_ (ng/mL or ng/g)*^a^	*AUC_0-tlast_ (ng•h/mL or ng•h/g)*^a^	T*_max_ (h)*
(A)			
Cornea	2.90 ± 1.70	15.5 ± 4.8	9.00
Aqueous humor	0.285 ± 0.166	1.45 ± 0.41	1.00
ICB	0.825 ± 0.505	5.12 ± 2.30	9.00
Upper eyelid margin	2,100 ± 410	8,500 ± 1,510	2.00
Lower eyelid margin	1,160 ± 340	6,220 ± 1,240	2.00
Bulbar conjunctiva	75.4 ± 21.9	517 ± 107	9.00
Periorbital fat	36.1 ± 10.8	139 ± 25.6	2.00
Retina (area centralis)	BLQ	NA	NA
(B)			
Cornea	52.5 ± 20.8	247 ± 57	9.00
Aqueous humor	3.29 ± 0.35	18.6 ± 2.6	9.00
ICB	3.30 ± 1.11	22.2 ± 7.8	9.00
Upper eyelid margin	27.5 ± 17.0	202 ± 67	4.00
Lower eyelid margin	29.3 ± 19.5	204 ± 74	4.00
Bulbar conjunctiva	137 ± 39	724 ± 230	9.00
Periorbital fat	1.97 ± 0.79	9.27 ± 2.53	9.00
Retina (area centralis)	BLQ	NA	NA

^a^ Data shown are mean ± standard error.

AUC_0-tlast_, area under the concentration–time curve from time zero to the last measurable time point; BLQ, below the limit of quantitation; *C*_max_, maximal observed concentration; ICB, iris–ciliary body; NA, not applicable; *T*_max_, time corresponding to maximal observed concentration.

Following a single administration of Bimatoprost SR 15 μg, bimatoprost and bimatoprost acid distributed primarily to the tissues adjacent to the implant (Table 2). The rank order of bimatoprost and bimatoprost acid exposure (AUC_0-tlast_) was as follows: ICB > cornea > aqueous humor > bulbar conjunctiva, where bimatoprost exposure in the bulbar conjunctiva was 300,000-fold lower than in the ICB. Neither bimatoprost nor bimatoprost acid was detectable in the eyelid margins, periorbital fat, or the area centralis of the retina. Very little metabolic conversion of bimatoprost to bimatoprost acid was observed in the ICB, cornea, and aqueous humor. In contrast, exposure to bimatoprost acid in the bulbar conjunctiva was 5.7-fold higher than exposure to bimatoprost.

**Table 2.** Pharmacokinetic Parameters of (A) Bimatoprost and (B) Bimatoprost Acid in Ocular Tissues Following the Administration of a Single Bimatoprost SR 15 μg to Beagle Dogs

*Ocular tissue*	C*_max_ (ng/mL or ng/g)*^a^	*AUC_0-tlast_ (ng•day/mL or ng•day/g)*^a^	T*_max_ (day)*
(A)			
Cornea	2,980 ± 530	93,800 ± 14,900	52
Aqueous humor	22.9 ± 19.6	727 ± 511	27
ICB	18,100 ± 16,700	486,000 ± 441,000	52
Upper eyelid margin	BLQ	NA	NA
Lower eyelid margin	BLQ	NA	NA
Bulbar conjunctiva	0.129 ± 0.055	1.62 ± 0.69	52
Periorbital fat	BLQ	NA	NA
Retina (area centralis)	BLQ	NA	NA
(B)			
Cornea	37.6 ± 8.57	1,300 ± 230	52
Aqueous humor	1.27 ± 0.30	44.0 ± 6.6	52
ICB	192 ± 186	2,420 ± 2,330	52
Upper eyelid margin	BLQ	NA	NA
Lower eyelid margin	BLQ	NA	NA
Bulbar conjunctiva	0.236 ± 0.149	9.25 ± 2.46	80
Periorbital fat	BLQ	NA	NA
Retina (area centralis)	BLQ	NA	NA

^a^ Data shown are mean ± standard error.

AUC_0-tlast_, area under the concentration–time curve from time zero to the last measurable time point; Bimatoprost SR, bimatoprost sustained-release implant; BLQ, below the limit of quantitation; *C*_max_, maximal observed concentration; ICB, iris–ciliary body; NA, not applicable; *T*_max_, time corresponding to maximal observed concentration.

### Comparison of intracameral and topical dosing

Bimatoprost concentrations higher than the half-maximal effective concentration of bimatoprost for contraction of the isolated feline iris sphincter (EC_50_, 14 ng/g^22^) were observed in the bulbar conjunctiva, eyelid margins, and periorbital fat following administration of topical bimatoprost 0.03% (Table 1). In comparison, concentrations of bimatoprost plus bimatoprost acid were below the LLOQ in the eyelid margins and periorbital fat following intracameral administration of Bimatoprost SR 15 μg (Fig. 3). Even in the bulbar conjunctiva, the *C*_max_ of bimatoprost plus bimatoprost acid was 600-fold lower following Bimatoprost SR 15 μg administration compared with topical treatment (Fig. 3).

Notably, high concentrations of bimatoprost plus bimatoprost acid were observed in the ICB (a site of pharmacologic action) and the aqueous humor (a surrogate used for measuring effective drug concentrations) following administration of Bimatoprost SR 15 μg (Fig. 4). In the ICB, the *C*_max_ for bimatoprost plus bimatoprost acid was 4,400-fold greater with Bimatoprost SR 15 μg (18,200 ng/g) than with topical bimatoprost 0.03% (4.13 ng/g).

## Discussion

This nonclinical study characterized the distribution of bimatoprost and bimatoprost acid in ocular tissues following intracameral administration of Bimatoprost SR 15 μg. Bimatoprost SR delivered bimatoprost and its acid metabolite to the ICB of dogs in a more selective and targeted manner than topical dosing with bimatoprost 0.03% ophthalmic solution. Following the administration of Bimatoprost SR 15 μg, low or undetectable bimatoprost and bimatoprost acid concentrations were observed in ocular tissues associated with PGA-related AEs, such as the bulbar conjunctiva, eyelid margin, periorbital fat, and retina. In contrast, with the exception of the retina (where there were no detectable concentrations of bimatoprost or bimatoprost acid with either administration method), comparatively high concentrations were observed in these tissues following topical bimatoprost 0.03% administration.

These nonclinical findings are consistent with recently reported 6-month interim results from a phase 1/2 clinical study in patients with open-angle glaucoma who received a single intracameral administration of Bimatoprost SR (6, 10, 15, or 20 μg) in the study eye and daily administration of bimatoprost 0.03% ophthalmic solution in the fellow eye.^20^ Ocular AEs that occurred in study eyes in the immediate postadministration period (within 2 days of Bimatoprost SR administration) generally resolved quickly and were likely related to the intracameral injection procedure.^20^ However, the overall incidence of PGA-associated AEs (defined as conjunctival hyperemia, eyelash growth, iris pigmentation, periorbital pigmentation, blepharitis, eyelid erythema, eyelid edema, and periorbital fat atrophy) with onset after 2 days was higher in the eyes treated with topical bimatoprost 0.03% [16 (21.3%) patients] than in those treated with Bimatoprost SR [7 (9.3%) patients].^20^ Importantly, no cases of periocular skin discoloration, periorbital fat atrophy, or eyelash growth were reported in eyes that received Bimatoprost SR.^20^

The results of this study confirm that Bimatoprost SR targets bimatoprost delivery to a site of action for IOP lowering and avoids bimatoprost exposure to eyelid, conjunctival, and periorbital tissues. Patients who experience PGA-associated AEs following the use of topical formulations may choose to discontinue glaucoma treatment.^23^ However, nonadherence to treatment puts patients at higher risk of visual field loss.^24^ Moreover, discontinuation of topical PGA treatment does not always result in rapid resolution of the AEs, as eyelid pigmentation changes, eyelash growth, and periorbital lipodystrophy (e.g., enophthalmos, periorbital fat atrophy, and deepening of the upper eyelid sulcus) may be only slowly reversible, and increased iris pigmentation may be irreversible.^25,26^

The results of this study also add to the growing body of evidence suggesting the potential value of sustained-release drug delivery systems for treating glaucoma. In a nonclinical study using Wistar rats, placement of a bimatoprost chitosan polymeric-based insert into the conjunctival sac was associated with sustained release of bimatoprost and decreased IOP.^27^ Additionally, a phase 1b clinical study in patients with elevated IOP found that 6 months of clinically significant IOP reduction could be achieved through the administration of a bimatoprost ring, a soft, flexible, ocular insert of bimatoprost in a silicone matrix that rests circumferentially in the fornices.^28^ No unexpected AEs were observed, and the most frequently reported ocular AEs were eye discharge and epiphora. A subsequent phase 2 study demonstrated similar decreases in IOP with the bimatoprost ring and timolol eye drops.^29^

The intracameral placement of Bimatoprost SR may lead to differences in IOP lowering and safety profile compared with the extraocular sustained-release drug delivery devices described above. In the phase 1/2 clinical study of Bimatoprost SR, a single implant reduced IOP reduction similarly to daily topical bimatoprost 0.03% administration for 3–4 months.^20^ These results are consistent with the drug distribution study in dogs presented in this study, which suggest that Bimatoprost SR provides targeted drug delivery to the ICB, the site of bimatoprost effects on IOP.^30^

In this study on dogs, peak levels of bimatoprost were present in the ICB at 51 days after Bimatoprost SR administration, and these levels were ∼4 log units higher than the peak levels of bimatoprost achieved by topical dosing. These results are consistent with the sustained decreases in IOP previously observed in dogs through at least 65 days after Bimatoprost SR administration^31^ and in humans in the phase 1/2 study of Bimatoprost SR.^20^ Interestingly, in the phase 1/2 study, effects of Bimatoprost SR persisted in many patients after the implant had degraded and drug levels would be expected to be negligible. At 2 years after a single administration of Bimatoprost SR, 28% of patients still had not required IOP-lowering rescue or retreatment with implant in the study eye.^32^ A possible explanation for this extended duration of effect might involve tissue remodeling. Topical administration of PGAs in cynomolgus monkeys leads to ciliary muscle remodeling, with enlargement of the elongated spaces between muscle bundles, which are the pathways for aqueous outflow.^33^ This remodeling is believed to be secondary to PGA-stimulated upregulation and release of matrix metalloproteases by ciliary muscle cells.^34^ It can be speculated that the sustained drug release and the very high ciliary body drug concentrations after Bimatoprost SR administration result in more durable tissue remodeling and longevity of the effect of Bimatoprost SR on IOP.

Species differences in the anatomy and physiology of the eye can result in differences in pharmacokinetic parameters after ocular drug administration.^35^ Importantly, the aqueous humor flow rate in dogs is approximately twice that in humans (4–6 μL/min vs. 2–3 μL/min, respectively^31^), and this increase in flow may contribute to more rapid drug release from implants in dog eyes. Therefore, the pharmacokinetics of bimatoprost after Bimatoprost SR administration in dogs may not translate to humans. In fact, in the 6-month interim results from the phase 1/2 clinical study, a single administration of Bimatoprost SR controlled IOP in 71% of human study eyes for up to 6 months.^20^ Nonetheless, the limited distribution of bimatoprost to the conjunctiva and eyelids after administration of Bimatoprost SR compared with topical dosing in dogs is consistent with the clinical trial results showing reduced incidence (after the immediate post-administration period) of AEs affecting the conjunctiva and eyelids in human eyes receiving Bimatoprost SR compared with topically treated fellow eyes. In addition, 2 patients in the clinical trial developed periorbital fat atrophy in the eye receiving bimatoprost drops, whereas the study eye treated with Bimatoprost SR showed no evidence of periorbital fat atrophy.^20^ These results are consistent with the dog pharmacokinetics data, which showed no detectable drug levels in this off-target extraocular tissue after Bimatoprost SR administration. The dog pharmacokinetics data suggest that Bimatoprost SR reduces the potential for common AEs associated with topical PGA treatment by limiting bimatoprost distribution to conjunctiva, eyelids, and periocular tissues.

Ocular drug metabolism may also differ across species. Although conversion of bimatoprost to its acid metabolite is limited in some species such as monkey,^36^ bimatoprost acid is present in corneal tissue and aqueous humor after topical bimatoprost administration in both dogs (this study) and humans.^37^ Thus a difference in bimatoprost metabolism is unlikely to affect the translatability of the results from dogs to humans.

The results of this study demonstrate that the extent of ocular metabolism of bimatoprost may be dependent upon the route of administration. The metabolite-to-parent drug AUC ratio in ocular tissues of dogs in this study was much lower following implant administration than following topical bimatoprost administration. For a PGA prodrug that is metabolized to the active compound, such as latanoprost,^38,39^ lack of drug metabolism could be associated with decreased efficacy. However, bimatoprost is active when unmetabolized.^39,40^

In this study, there was a selection bias for including animals with larger iridocorneal angles in the Bimatoprost SR treatment group, because of a greater possibility of the implant contacting the corneal endothelium in smaller angles. Therefore, the lack of evaluation of the effects of angle size on drug distribution is a study limitation. Nevertheless, it is unlikely that differences in angle size between the Bimatoprost SR and topical bimatoprost treatment groups affected the results, because the baseline IOPs and the remainder of the ocular anatomy were similar between groups.

In summary, the data presented herein suggest that treatment of glaucoma patients with Bimatoprost SR may reduce the incidence of side effects typically associated with the administration of topical PGAs by targeting bimatoprost delivery to the key site of action of the PGA class and reducing bimatoprost exposure to off-target ocular tissues.
